# Bio-Based Polyurethane Foams for the Removal of Petroleum-Derived Pollutants: Sorption in Batch and in Continuous-Flow

**DOI:** 10.3390/polym15071785

**Published:** 2023-04-03

**Authors:** Fabrizio Olivito, Vincenzo Algieri, Antonio Jiritano, Matteo Antonio Tallarida, Paola Costanzo, Loredana Maiuolo, Antonio De Nino

**Affiliations:** Department of Chemistry and Chemical Technologies, University of Calabria, Via P. Bucci, Cubo 12C, 87036 Rende, CS, Italy; vincenzo.algieri@unical.it (V.A.); antonio.jiritano@unical.it (A.J.); matteoa.tallarida@unical.it (M.A.T.); paola.costanzo@unical.it (P.C.); maiuolo@unical.it (L.M.)

**Keywords:** bio-based polyurethanes, petroleum pollutants, batch sorption, continuous-flow, regeneration

## Abstract

In this paper, we evaluated the potential of two synthesized bio-based polyurethane foams, PU1 and PU2, for the removal of diesel and gasoline from water mixtures. We started the investigation with the experiment in batch. The total sorption capacity S (g/g) for the diesel/water system was slightly higher with respect to gasoline/water, with a value of 62 g/g for PU1 and 65 g/g for PU2. We found that the sorption follows a pseudo second-order kinetic model for both the materials. The experimental data showed that the best isotherm models were obtained with Langmuir and Redlich–Peterson models. In addition, to provide an idea of the process scalability for future industrial applications, we tested the sorption capacity of the foams using a continuous-flow of the same oil/water mixtures and we obtained performances even better with respect to the batch test. The regeneration can be performed up to 50 times by centrifuge, without losing efficacy.

## 1. Introduction

Despite efforts made to replace fossil fuels with eco-sustainable and renewable alternatives, they are still used worldwide as a primary resource for energy production [1,2]. Over 80% of the produced energy comes from non-renewable sources [3]. The European Green Deal has set the goal to make Europe the first climate-neutral continent by 2050, but the route to the transition still seems long and complicated [4]. Meanwhile, petroleum-derived products in quantities equal or higher than 5 million tons are transported overseas every year [5]. Large or small accidents involving fuel transportation generate permanent damage to the ecosystem [6,7]. Refineries have been moved closer to large population centers over the years to avoid transportation, but this has led to the bio-accumulation of petroleum-derived hydrocarbons in the underground and surface waters [8]. Still, for several years there will be the need to develop new methods for the removal of these dangerous pollutants from water sources. Oil contaminants are commonly detected in the form of fats, lubricants and light and heavy hydrocarbons such as gasoline, kerosene and diesel [9,10]. There are different methodologies that have been introduced to decontaminate water sources, and they can be divided into three macro-areas: chemical, physical and biological [11,12,13]. In this paper, we focused our attention on sorption and the chemical–physical parameters that affect this methodology [14]. Sorbent can be divided into two categories: synthetic and natural [15,16]. Synthetic sorbents such as rubbers, membranes, foams and aerogels are generally made from non-renewable chemicals, and they possess low or no tendency to biodegradation in natural conditions [17,18]. These materials, despite the environmental concerns, have also some advantages, such as reduced production costs, mechanical resistance, high adsorption capacity and reusability [19]. Given the growing awareness of finding an alternative to the finite resources of this planet, several renewable raw materials have been introduced over the years for the production of materials or composites [20,21,22]. Among the most important examples are polylactic acid, polycaprolactone, derivatives of chitosan, starch and cellulose [23,24,25,26].

Among the natural sorbent, cellulose was used in recent years to remove different kinds of pollutants from waters such as hydrocarbons, dyes, heavy metals and others because of both its hydrophobic core and its hydrophilicity due to the hydroxyl groups [27,28,29]. Cellulose and its derivatives can also be used as additives or reagents in polymerization reactions to replace non-renewable chemicals and create bio-based materials [30,31,32]. Bio-based materials not only have the advantage of being cheaper because they exploit renewable resources, but also possess biodegradability and eco-compatibility characteristics [33,34].

Polyurethanes are a class of versatile materials that are widely used for environmental purposes [35]. They are generally made from fossil resources, but the introduction of new renewable chemicals in the production process represents the new era of this topic [36,37]. However, synthetic polyurethanes possess extraordinary stability and a low tendency to be degraded by natural and atmospheric agents [38]. The introduction of natural feedstocks can provide a solution to the biodegradability without losing the most important chemical–physical properties [39]. In our previous work, we synthesized two new types of soft-polyurethane foams by employing a cellulose-derived polyol for the chain extension of the prepolymer and the same chain extender mixed with cellulose citrate used as thickener additive [40,41].

In this work, we proved that these bio-based polyurethanes are efficient to remove petroleum-derived pollutants from artificially contaminated water samples. We obtained high adsorption capacity both for diesel and gasoline contaminated samples, and these foams can be reused after a simple centrifugation up to 50 times. Finally, to prove the scalability of the process at the industrial level, we evaluated the sorption capacity by passing the same oil/water mixtures in continuous-flow. The results showed an improvement in the sorption capacity with respect to the batch test, confirming the wide applicability of this new type of bio-based materials.

## 2. Materials and Methods

### 2.1. Chemicals

Tetrahydrofuran (THF) was purchased from Carlo Erba (Milan, Italy) at analytical grade and freshly distilled before use after drying over sodium sulfate. Acetone was purchased from Honeywell at a high purity grade and used without further purification. Microcrystalline cellulose type 102 was purchased from Roquette (Lestrem, France) at high purity. Citric acid was purchased from Sigma Aldrich (St. Louis, MO, USA) at 99% purity grade. Sodium borohydride was purchased from Carlo Erba (Milan, Italy) at 95% purity grade. Iodine was purchased from Carlo Erba (Milan, Itay) at the analytical grade. Polyethylene glycol (PEG) 400 was purchased from Thermo Fisher Scientific (Waltham, MA, USA) at 99% purity grade. The 2,2,4-Trimethylhexamethylene diisocyanate (TMDI) was purchased from Evonik Industries (Essen, Germany), at 95% purity grade. Sodium chloride and sodium sulfate anhydrous was purchased from Sigma Aldrich (St. Louis, MO, USA) at analytical grade. IR spectra and SEM images of all products are reported in reference [41].

### 2.2. Synthesis of Bio-Oil and Cellulose Citrate from Microcrystalline Cellulose

Bio-oil and cellulose citrate were prepared by the same reaction described elsewhere [40]. Microcrystalline cellulose (10 g) was mixed with citric acid (10 g) (monohydrate or anhydrous) in equivalent weight ratio, in an open-air Pyrex flask, and the system was heated to 154 °C. After 2 h, the mixture was cooled down and washed several times with acetone to extract the bio-oil adsorbed onto the solid. Acetone was removed under reduced pressure to get bio-oil in the form of brown viscous oil. The collected yellow solid in the form of cellulose citrate was further washed with distilled water and dried in an oven at constant temperature of 70 °C for overnight time.

### 2.3. Bio-Oil Reduction into Polyol Chain Extender

Polyol chain extender was prepared by our reaction described elsewhere [41]. A quantity of 50 g of bio-oil was dissolved in 200 mL of THF dry in a 500 mL one-neck round bottom flask and 12.5 g (25% in weight respect to bio-oil) of NaBH_4_ was added portion-wise to this solution. After one hour, 15 g (30% in weight respect to bio-oil) of iodine was added slowly, and, after another hour, the excess NaBH_4_ was quenched with aqueous HCl 1 M. The mixture was filtered through a sintered glass funnel. The solution was dried over sodium sulphate and filtered, and the solvent was removed under vacuum to obtain a polyol in the form of a brown oil, with a yield of 85–90%.

### 2.4. Prepolymer Synthesis

Prepolymer was synthesized by our reaction described elsewhere [34]. The 2,2,4-Trimethylhexamethylene diisocyanate (TMDI) was used in a molar excess of 2.5:1 with respect to PEG 400. Water was used as a blowing agent at a weight percentage of 5.6% with respect to PEG 400. The reagents were added in the following order in a plastic container: PEG 400, distilled water and NaCl as catalyst. The mixture was stirred using a mechanical apparatus. The diisocyanate was added and the mixture was vigorously stirred. The blend was warmed up to 70 °C for one hour until diisocyanate consumption. The prepolymer was obtained using the same procedure in the form of a colorless gel.

### 2.5. Synthesis of PU1 and PU2

Polyurethanes PU1 and PU2 were prepared by our reaction described elsewhere [41]. Polyol chain extender was added to the freshly prepared prepolymer at 30% weight with respect to the prepolymer, and the mixture was vigorously and mechanically stirred for a few minutes in the same plastic container. After that, the same mixture was transferred into a steel mold, and the system was closed under pressure at room temperature. After eight hours, the polyurethane foam PU1 was obtained in the final form.

Polyol chain extender at 30% weight and cellulose citrate at 20% weight with respect to the prepolymer were added sequentially to the same plastic container as the prepolymer, and the mixture was vigorously and mechanically stirred for a few minutes. After that, the same mixture was transferred into a steel mold, and the system was closed under pressure at room temperature. After eight hours, the polyurethane foam PU2 was obtained in the final form.

**Figure 1:**
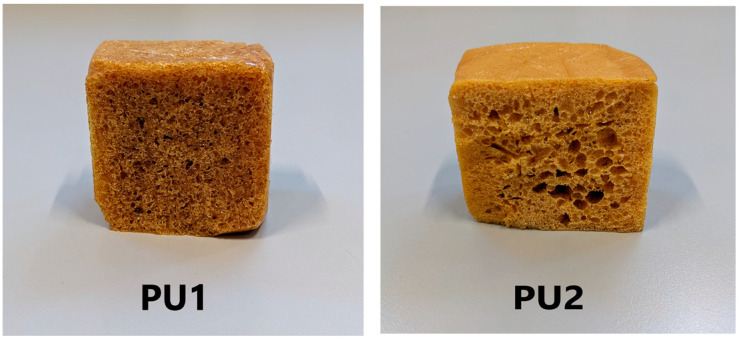
Polyurethane foams PU1 and PU2.

PU1 and PU2 are reported in the Figure 1.

### 2.6. Sorption Capacity Test in Batch Using Oil/Water System

The sorption capacity (S) in oil/water system in batch conditions was determined as reported elsewhere [42]. The sorbent was cut in cubic form (1 g) and put in the oil/water mixture at the concentration defined in this work. Distilled water was used to prepare the mixtures. The oil/water mixture was poured into a 2 L beaker, and, after the addition of the sorbent, the system was stirred at room temperature through a magnetic stir bar and a stirring plate at 50 rpm for the time defined in this work. After the defined time, the material was removed and placed in a centrifuge using a conical bottom centrifuge tube to collect the oil (Vivaspin^TM^ 6 centrifugal concentrators, purchased from Fischer Scientific, Waltham, MA, USA). The sorbed materials were centrifuged at 500 rpm with a rotor radius of 11 cm for 1 min until the oil was separated on the bottom. The regeneration can be repeated up to 50 times. The collected biphasic system consisting of oil with a small percentage of water was subjected to liquid/liquid extraction, using three portions of petroleum ether to extract the organic phase. The organic phases were collected and dried over sodium sulphate anhydrous. The mixture was filtered using a sintered glass funnel in a one-neck round bottom flask and the solvent removed under reduced pressure. The oil sorption capacity was calculated by the following Equation (1):

(1) $$S(g/g)=\frac{M_{t}-M_{0}}{S_{0}}$$

where M_0_ is the weight of the reaction flask, M_t_ is the weight of the reaction flask plus the collected oil after the process and S_0_ is the initial weight of the sorbent.

### 2.7. Sorption Capacity Test in Continuous-Flow Using Oil/Water System

The same oil/mixtures used for batch test were used also in this case. We used a peristaltic pump connected to a glass cartridge in which the solution is passed continuously in up-flow mode and the solution collected at the end of the system. The two foams were inserted in the cartridge and fixed above and below with glass balls to avoid movement of the sorbent during the flow. The process is divided into two phases: the first phase of sorption in which the oil/water solution is passed through the cartridge, while the second phase consists of desorption in which diethyl ether is used to desorb oil. The organic phase is added to water and extracted three times, dried on sodium sulfate and the solvent removed under vacuum.

**Figure 2:**
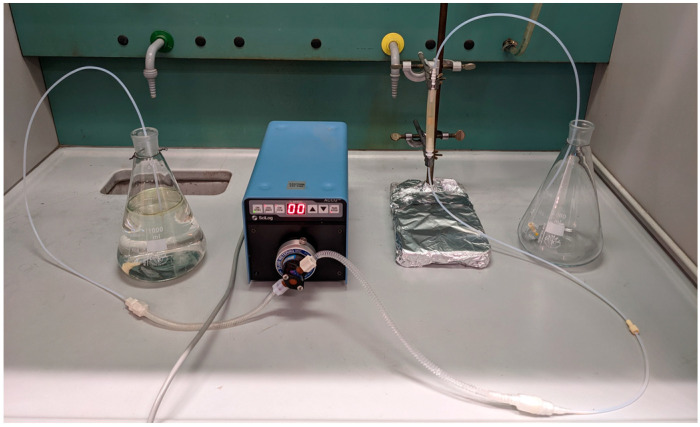
Apparatus used for the sorption in continuous flow.

The employed apparatus is illustrated in Figure 2.

## 3. Results and Discussion

Our aim was to test two renewable polyurethane foams, PU1 and PU2, for water purification from petroleum-derived pollutants. In Table 1, we reported some of the physical properties of the two foams, PU1 and PU2, estimated by SEM in our previous work [41].

The materials PU1 and PU2 have an anisotropy index close to 1, which proves the spherical shape of the cells. In addition, the number of cells is higher for PU2, probably due to the fact that the addition of cellulose citrate inside the polymer chains promotes the formation of more pores. In contrast, the average cell area is lower due to the fact that cellulose citrate, although it promotes the formation of more pores, fills the porous cavities.

In the first part, we evaluated through batch experiments the reusability of PU1 and PU2 with the relative total sorption capacity. We also investigated the sorption mechanism through the analysis of the kinetic of oil sorption in gasoline/water and diesel/water systems until the equilibrium is reached. Pseudo first-order and pseudo second-order models were evaluated because they are the most-employed kinds of these materials [43,44]. Then we tested the sorption capacity using different concentrations of oils, and the collected experimental data were compared with the common adsorption isotherm models (Langmuir, Freundlich). The total sorption capacity was compared with the data available from the recent literature. At the end, to give a more realistic view of the possibility to scale-up the process, we performed the experiment in continuous-flow, and we calculated the total sorption capacity in the same oil/mixtures of the two foams, making a comparison with batch tests.

### 3.1. Total Sorption Capacity of PU1 and PU2 in Gasoline/Water and Diesel/Water Systems in Batch

**Figure 3:**
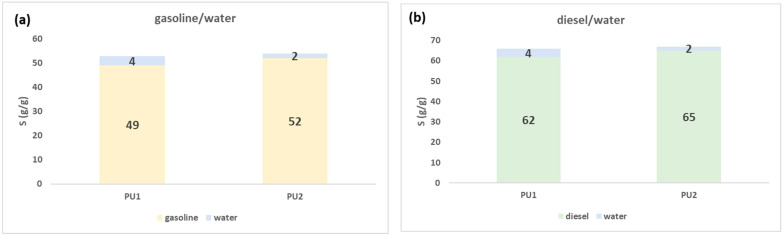
Total sorption capacity of PU1 and PU2 in gasoline/water (**a**) and diesel/water (**b**) starting from a solution of 80 g/L.

We evaluated the total sorption capacity S (g/g) of PU1 and PU2 starting from 80 g/L solution of gasoline/water and diesel/water systems. After the first tests, we found that both materials reached the equilibrium of sorption after one minute. Due to the instantaneous process, simultaneous sorption is more likely than competitive sorption. Regeneration can be performed up to 50 times by centrifuging PU1 and PU2 after the batch test. The sorption capacity remains unchanged after the centrifugation cycles, without the material undergoing deformation or flaking. In Figure 3, we reported the plots of S (g/g), with the quantity of sorbed oil and water. Water and oil can be easily separated by the method described in Section 2.5.

From the data shown in Figure 3, we obtained the confirmation of the high hydrophobicity of these materials because both in gasoline/water and diesel/water systems the quantity of water sorbed ranged from 2 to 4%. The sorbed gasoline was 49 g/g and diesel 62 g/g for PU1 while the sorbed gasoline was 52 g/g and diesel 65 g/g for PU2. The affinity of PU1 and PU2 is slightly higher for diesel than gasoline; this is probably due to the higher density of diesel with respect to gasoline, which allows a better retention in the pores of the polymers [45,46].

### 3.2. Sorption Kinetics of PU1 and PU2 in Gasoline/Water and Diesel/Water Systems in Batch

Assuming that pseudo first-order and second-order models are the most appropriate for this kind of study, we evaluated the sorption kinetics, and the relative equations are reported as follows:

(2) $$\ln\;\frac{q_{e}}{q_{e}-q_{t}}=k_{1}t$$

(3) $$\frac{t}{q_{t}}=\frac{1}{q_{e}t}+\frac{1}{k_{2}q_{e}^{2}}$$

where q_e_ is the maximum oil sorbed at the equilibrium, q_t_ is the oil sorbed at the time t and k_1_ and k_2_ are the first and second order rate constants.

In the performed tests, we found that the equilibrium was reached after one minute for both the foams. In Table 2, we reported the experimental and theoretical q_e_ (g/g) values and the relative kinetic parameters of PU1 and PU1 for the sorption of gasoline and diesel. The experimental q_e_ was obtained from batch tests.

**Figure 4:**
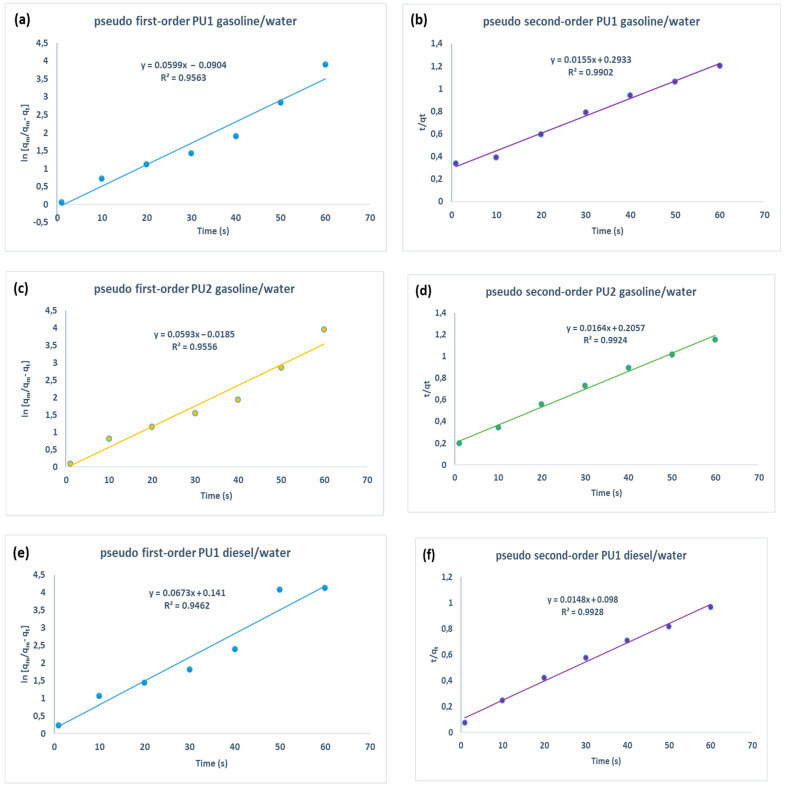

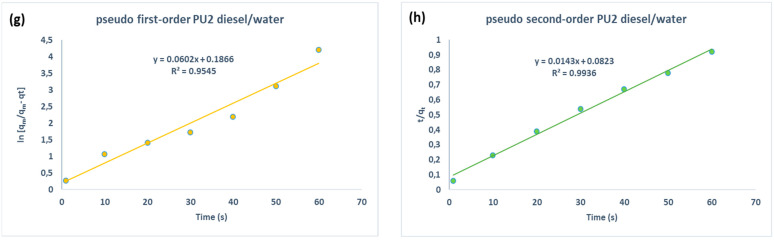
Pseudo first-order (**a**) and pseudo second-order (**b**) sorption linear fitting of PU1 in gasoline/water system. Pseudo first order (**c**) and pseudo second-order (**d**) sorption linear fitting of PU2 in gasoline/water system. Pseudo first-order (**e**) and pseudo second-order (**f**) fitting of PU1 in diesel/water system. Pseudo first order (**g**) and pseudo second-order (**h**) fitting of PU2 in diesel/water system.

We used pseudo-first order, pseudo-second order and intraparticle diffusion models to rationalize the sorption mechanism. The first model describes processes with the following characteristics: linear equilibrium of sorption, dependency by time but not by solute concentration, systems which quickly reach equilibrium and so on [46,47]. These situations often occur in the initial stages of the sorption process. The second one is the ideal system to describe processes in which chemical interactions represent the driving force with respect to other purely physical processes, such as inclusion, occlusion and diffusion [48,49]. The intraparticle diffusion model is not the rate controlling step for this kind of process because the plot is not linear by the analysis of the correlation coefficients and does not pass through origin and C_i_ ≠ 0 as reported in Table 2 and Supporting Information. We reported in Figure 4 the kinetic plots relative to the sorption of polyurethane PU1 and PU2 to make a comparison between pseudo first-order and pseudo second-order.

The analysis of the correlation coefficients in Table 1 and the plots in Figure 3 proves that the pseudo second-order kinetic model is the best fit to describe the data collected using PU1 and PU2 both in gasoline/water and diesel/water systems. Generally, the rate-limiting step of pseudo second-order model is the surface adsorption through chemisorption, which involves chemical interactions rather than physical ones [50]. Therefore, chemical interactions are the major driving forces at the base of the sorption mechanism of this study. The two materials have a slightly greater affinity for the diesel phase with respect to gasoline. The major difference between the two materials lies in the maximum quantity of oil sorbed at the equilibrium q_e_, obtained from the experimental batch test. This can probably be rationalized by the fact that the insertion of cellulose citrate inside the polymer raises the hydrophobicity and therefore the affinity itself towards these petroleum-hydrocarbon pollutants. In addition, the experimental q_e_ is higher for diesel than gasoline for both materials probably due to diesel viscosity, which allows it to be more retained in the pores of the polyurethanes [46].

### 3.3. Adsorption Isotherms in Gasoline/Water and Diesel/Water Systems in Batch

For a deeper understanding, we investigated which was the best isotherm model to represent the obtained experimental data among Langmuir, Freundlich and Redlich–Peterson models in gasoline/water and diesel/water system. Langmuir isotherm is a model relating to a type of process which consists of a monolayer adsorption on a homogeneous surface in which the functional groups are also distributed equally and no chemical reaction occurs between the adsorbent and the adsorbate [46]. The equation relative to the first model is the following:

(4) $$q_{e}=q_{max}\frac{K_{L}C_{e}}{1+K_{L}C_{e}}$$

in which q_max_ is the maximum sorption capacity (g/g), q_e_ is the quantity of oil sorbed at the equilibrium (g/g), C_e_ is the oil concentration at the equilibrium (g/L) and K_L_ is the Langmuir isotherm constant (L/g). In addition, we calculated the dimensionless separation factor (R_L_), a commonly used parameter that can be calculated by using the Langmuir parameter K_L_ [51]_._ The equation is the following:

(5) $$R_{L}=\frac{1}{1+K_{L}C_{0}}$$

in which K_L_ (L/g) is the Langmuir constant and C_0_ is the initial oil concentration (g/L). This value is important because it estimates whether the process is unfavorable (R_L_ > 1), linear (R_L_ = 1), favorable (0 < R_L_ < 1) or irreversible (R_L_ = 0) [52].

The Freundlich isotherm models are related to non-ideal adsorption on a heterogeneous surface in which the process occurs on multilayer instead of monolayer. It is represented by the following equation:

(6) $$q_{e}=K_{F}C_{e}^{1/n}$$

in which K_F_ represents the Freundlich equilibrium constant related to adsorption capacity, *n* represents the affinity of the oil for the sorbent material and q_e_ is the unit weight of oil sorbed at the equilibrium for unit weight of the sorbent material [52].

The last model employed in this study is the Redlich–Peterson model, useful in this case because it generally describes the adsorption of heavy metals or generic organic matter as petroleum-derived pollutants. It is a combination of Langmuir and Freundlich models and for these reasons it presents some advantages. Here, we report the equation with the three characteristic parameters: A, B and β [52].

(7) $$q_{e}=\frac{AC_{e}}{1+BC_{e}^{\beta }}$$

in which A is the quantity of A in liters per gram of sorbent (L/g) and B is the quantity of B in liters per gram of oil (L/g). When β is low (β < 1), and A and B > 1, this model is similar to the Freundlich model and A/B and (1 − β) are related to K_F_ and n.

When β = 1, the model is similar to the Langmuir model [52].

**Figure 5:**
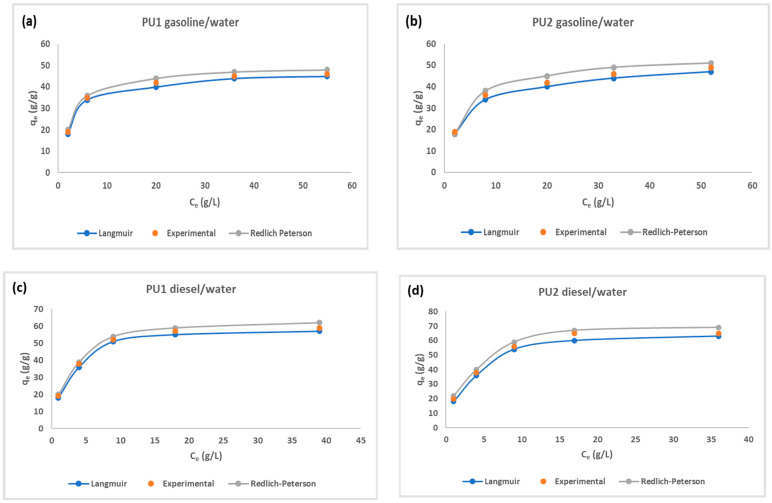
Langmuir and Redlich–Peterson models for the sorption of gasoline onto PU1 and PU2 (**a**,**b**) and for the sorption of diesel onto PU1 and PU2 (**c**,**d**).

We investigated the sorption of PU1 and PU2 in gasoline/water system and diesel/water system using different initial concentration: 40, 60, 80, 100 and 120 g/L. The best plots relative to the Langmuir and Redlich–Peterson models, together with the experimental data, are reported in the Figure 5.

The plots reported in Figure 5 and the relative correlation coefficients R^2^ of Table 3 proved that the best models are Langmuir and Redlich–Peterson. Langmuir model is relative to monolayer adsorption on a surface with a finite number of functional groups that are organized in a homogeneous manner. The Redlich–Peterson model furnished a good fit by the analysis of correlation coefficient and the value of β that is close to 1, so the model is similar to the Langmuir type [53]. For this reason, the sorption mechanism is also regulated by capillary forces other than surface sorption. Freundlich model did not furnish good correlation coefficients, proving that this model is more suitable for multilayer and heterogeneous adsorption (see Table 3 and Supplementary Materials). We reported the obtained parameters of the three models in Table 3.

From Table 3, the maximum sorption capacity q_m_ values obtained from the Langmuir equation were almost in accordance with experimental values obtained from the batch tests, and the same was obtained for Langmuir constant (K_L_), related to the sorption energy or the affinity of the material for the oil, which is higher for PU2 than PU1. This trend of the Langmuir model is in accordance with the experimental results both in gasoline/water and diesel/water systems.

One of the essential characteristics of the Langmuir isotherm can be expressed by a dimensionless constant, separation factor, R_L_.

The value of R_L_ indicates the type of the isotherm, which is unfavorable (R_L_ > 1), linear (R_L_ = 1), favorable (0 < R_L_ < 1) or irreversible (R_L_ = 0) [54].

For PU1, the range is 0.03 < R_L_ < 0.13 in gasoline/water and 0.01 < R_L_ < 0.08 in diesel/water systems, while, for PU2, the range is 0.02 < R_L_ < 0.12 in gasoline/water and 0.01 < R_L_ < 0.05 in diesel/water systems. These data represent another confirmation that the Langmuir isotherm is favorable for this process.

### 3.4. Comparison of the Total Sorption Capacity with Literature Data

We proved that PU1 and PU2 furnished optimal values of sorption capacity (S) in gasoline/water and diesel/water systems. In Table 4, we reported a comparison between S (g/g) values obtained in this study with the data collected from the recent literature.

From the data collected in Table 4, we proved by a comparison that these materials have provided excellent affinity with petroleum-derived pollutants from contaminated water samples. These values are higher compared to the recent literature and can be considered competitive with respect to common commercial synthetic materials.

### 3.5. Total Sorption Capacity of PU1 and PU2 in Gasoline/Water and Diesel/Water Systems in Continuous-Flow

**Figure 6:**
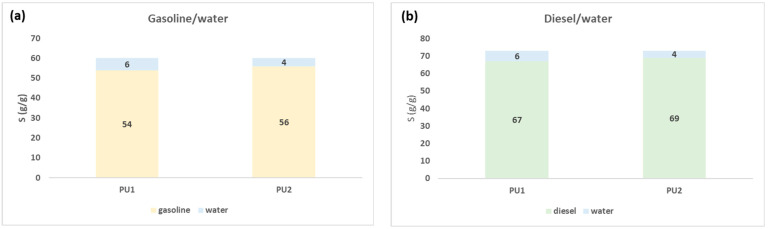
Total sorption capacity of PU1 and PU2 in gasoline/water (**a**) and diesel/water (**b**) systems starting from a solution of 80 g/L in continuous flow.

We decided to conclude this experimental work with the calculation of the total sorption capacity in a continuous-flow process, making a comparison with the batch experiment. We used solutions of gasoline/water and diesel/water, with a concentration 80 g/L, the same for the batch experiment. For this type of test, to evaluate the reusability of the materials, we did not use centrifuge to collect the sorbed oil, but we performed a desorption cycle using diethyl ether, a low boiling point solvent. Moreover, in this case, the regeneration can be performed up to 50 times with similar values of sorption capacity. The foams PU1 and PU2 have not undergone any decomposition or deformation by the solvent, maintaining the same affinity towards these pollutants. In Figure 6, we reported the obtained values of the total sorption capacity.

The total sorption capacity S (g/g) of PU2 is also higher with respect to PU1 both in gasoline/water and diesel/water systems in continuous-flow. It is relevant that the values of S are higher with respect to the values obtained in batch experiments. In this case, the total volume is forced to pass over the entire surface of the sorbent materials; therefore, the contact of the oily phase with the polyurethanes is more favored than in the batch experiments in which both the oil and the sorbing material are more dispersed with consequently less contact between them.

## 4. Conclusions

In this study, we evaluated the sorption capacity towards petroleum-derived pollutants of two bio-based polyurethane foams produced employing cellulosic materials. The two foams, PU1 and PU2, were initially tested in gasoline/water and diesel/water systems in batch. We obtained excellent results, with a slightly higher performance for PU2. The collected oil phases can be desorbed by a centrifuge, and the regeneration can be performed up to 50 times without losing the sorption capacity. We investigated the mechanism by kinetic and isotherm models. The process follows pseudo second-order model for both the materials in gasoline/water and diesel/water mixtures by obtaining excellent correlation coefficients. For the adsorption isotherm, we found that Langmuir model is appropriate to describe this process through a monolayer and homogeneous adsorption on a surface. By a comparison with the recent literature data in this field, we showed that the obtained results can be considered a significant advance. In addition, we evaluated the total sorption capacity using a continuous-flow process obtaining a better performance with respect to batch tests for both the materials. This final experiment may provide an idea as to the future scalability of the process in more real situations.

## Figures and Tables

**Table 1:** Main physical properties of the polyether polyurethane foams PU1 and PU2.

PU	Anisotropy Index ^a^	Number of Cell/(mm^2^)	Average Cell Area (mm^2^)
PU1	1.07 ± 0.06	10 ± 2	0.513 ± 0.063
PU2	1.14 ± 0.09	14 ± 2	0.361 ± 0.052

^a^ estimated by SEM.

**Table 2:** Maximum oil sorption capacities at equilibrium, rate constants and correlation coefficients evaluated at 25 °C.

**PU1**	**Gasoline**		**Diesel**
q_e_ (g/g)_experimental_	49.8		62.1
Pseudo first-order	R^2^	0.9563	0.9462
	q_e_ (g/g)	1.23	1.15
	k_1_	0.0599	0.0673
Pseudo second-order	R^2^	0.9902	0.9928
	q_e_ (g/g)	64.5	67.6
	k_1_	0.0155	0.0148
Intraparticle diffusion	R^2^	0.7273	0.8402
	C_i_	18.5	13.8
	k_i_	7.9	5.0
**PU2**	**Gasoline**		**Diesel**
q_e_ (g/g)_experimental_	51.9		65.2
Pseudo first-order	R^2^	0.9556	0.9545
	q_e_ (g/g)	1.12	1.20
	k_1_	0.0593	0.0602
Pseudo second-order	R^2^	0.9924	0.9936
	q_e_ (g/g)	60.9	69.9
	k_2_	0.0164	0.0143
Intraparticle diffusion	R^2^	0.8136	0.868
	C_i_	14.3	13.9
	K_i_	4.8	4.9

**Table 3:** Regression analysis of oil sorption by PU1 and PU2. Parameters estimated using Langmuir, Freundlich and Redlich–Peterson models.

**Gasoline/Water**	**PU1**		**PU2**
**Isotherm**			
Langmuir model	K_L_	0.33	0.38
	q_m_ (g/g)	47.3	49.7
	R^2^	0.9995	0.9975
Freundlich model	K_F_	17.39	15.44
	N	3.83	3.24
	R^2^	0.8756	0.9284
Redlich–Peterson model	A (L/g)	4.172	4.383
	B (L/g)^β^	0.077	0.081
	Β	1.124	1.125
	R^2^	0.9998	0.9909
**Diesel/water**	**PU1**		**PU2**
**Isotherm**			
Langmuir model	K_L_	0.60	0.96
	q_m_ (g/g)	59.5	63.3
	R^2^	0.9981	0.9971
Freundlich model	K_F_	20.29	19.99
	N	2.79	2.68
	R^2^	0.9698	0.9407
Redlich–Peterson model	A (L/g)	5.248	5.583
	B (L/g)^β^	0.097	0.103
	Β	1.135	1.136
	R^2^	0.9963	0.9942

**Table 4:** A comparison of the total oil sorption capacity (S) with the recent literature data in batch experiments.

Material	Oil	Sorption Capacity (g/g)	Reference
PU-LMA microsphere	Diesel	28.39	[42]
PU-g-LMA	Diesel	37.64	[42]
PU-ac	Diesel	32.5	[45]
	Gasoline	32.0	
PU-palm fiber	Diesel	28.9	[55]
PU-graphite	Diesel	20.0	[56]
Biochar-PDMS nanohybrid embedded PU sponge	Diesel	26.88	[57]
ZnO-PA sponge	Diesel	33	[58]
3D PU	Gasoline	15	[59]
Cellulosic aerogel	SF crude oil	57	[60]
PU1	Gasoline	49.8	This study
	Diesel	62.1	
PU2	Gasoline	51.9	This study
	Diesel	65.2	

## Data Availability

Not applicable.

## Supplementary Materials

The following supporting information can be downloaded at: https://www.mdpi.com/article/10.3390/polym15071785/s1. Figure S1: Freundlich plot for PU1 in gasoline/water; Figure S2: Freundlich plot for PU2 in gasoline/water system; Figure S3: Freundlich plot for PU1 in diesel/water; Figure S4: Freundlich plot for PU2 in diesel/water; Figure S5: Intra-particle diffusion plot for PU1 in gasoline/water; Figure S6: Intra-particle diffusion plot for PU2 in gasoline/water. Figure S7: Intra-particle diffusion plot for PU1 in diesel/water. Figure S8: Intra-particle diffusion plot for PU2 in diesel/water.
