# Supplementary material for: Bio-Based Polyurethane Foams for the Removal of Petroleum-Derived Pollutants: Sorption in Batch and in Continuous-Flow
Source: Polymers (Basel). 2023 Apr 3;15(7):1785. doi: 10.3390/polym15071785 (PMC10098679; doi:10.3390/polym15071785)
Supplement: Supplementary file 1 [file polymers-15-01785-s001.zip › polymers-2312675-supplementary.pdf]

FREUNDLICH MODEL PLOTS

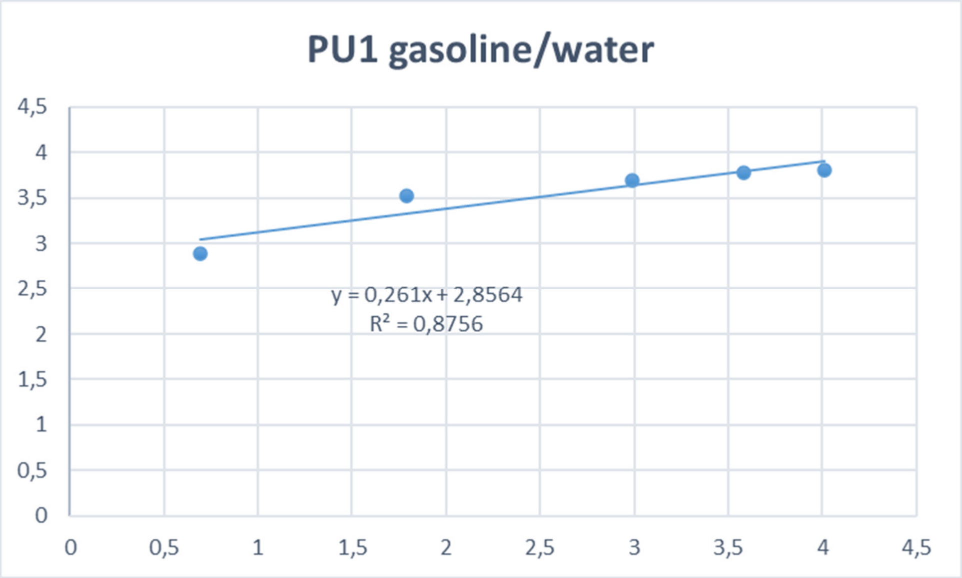

Figure S1. Freundlich plot for PU1 in gasoline/water.

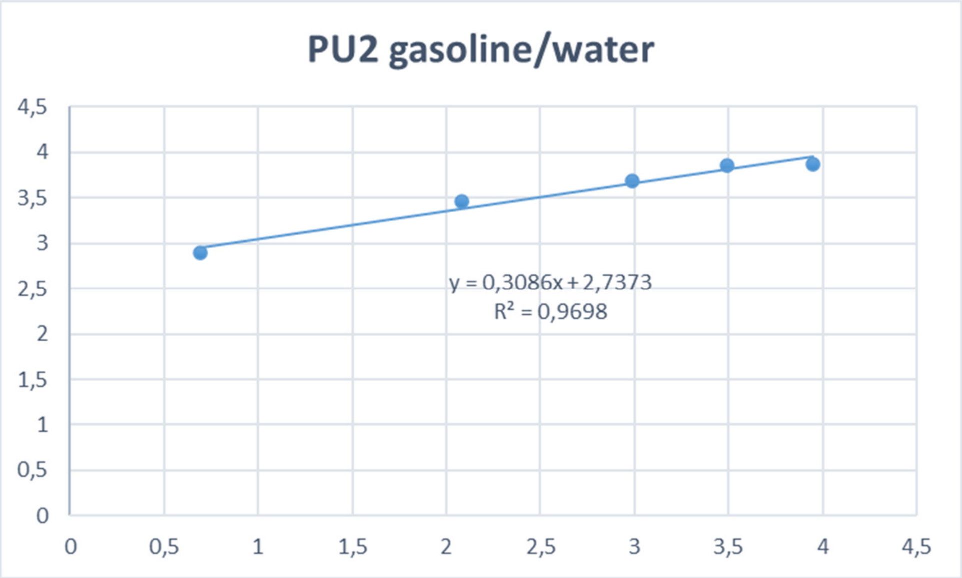

Figure S2. Freundlich plot for PU2 in gasoline/water system.

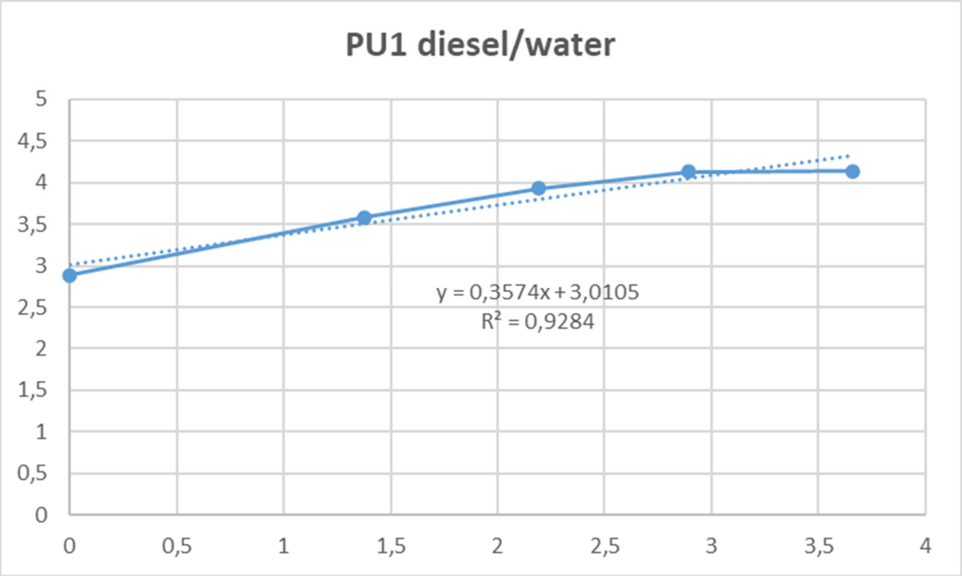

Figure S3. Freundlich plot for PU1 in diesel/water.

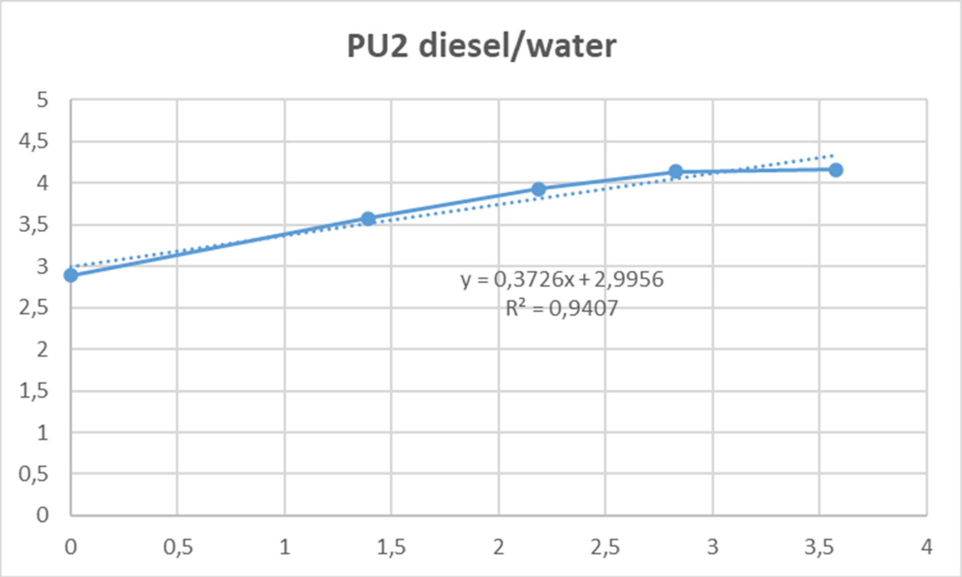

Figure S4. Freundlich plot for PU2 in diesel/water.

INTRA-PARTICLE DIFFUSION MODEL PLOTS

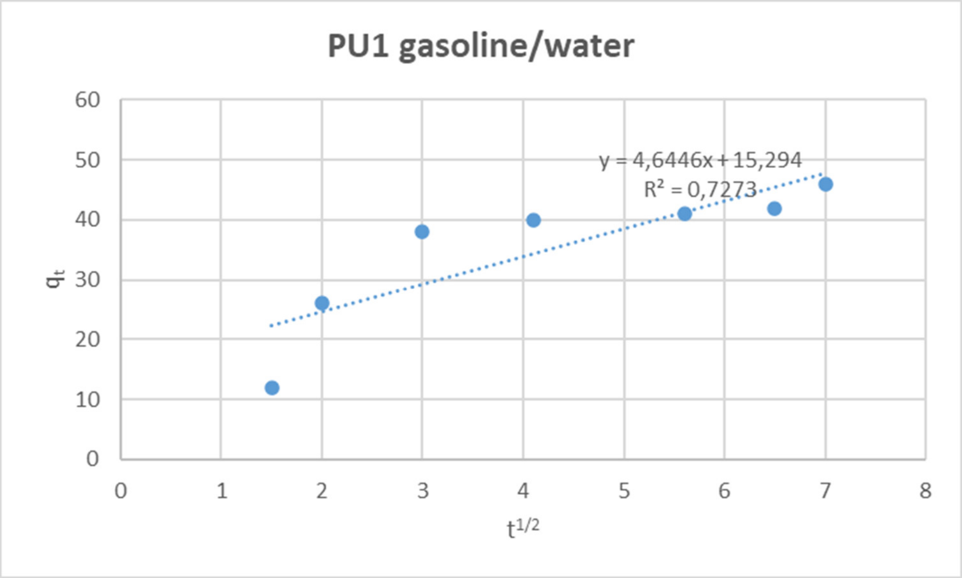

Figure S5. Intra-particle diffusione plot for PU1 in gasoline/water.

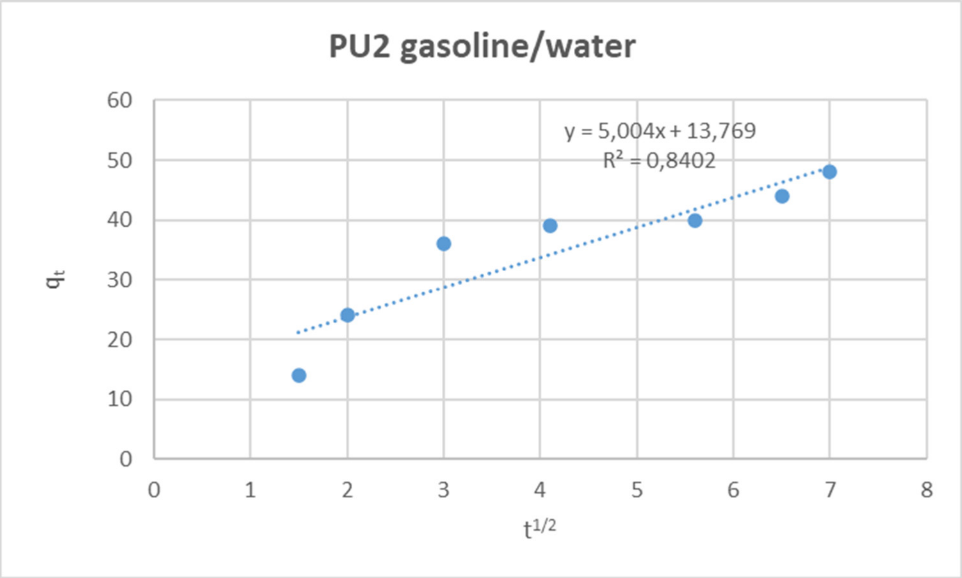

Figure S6. Intra-particle diffusion plot for PU2 in gasoline/water.

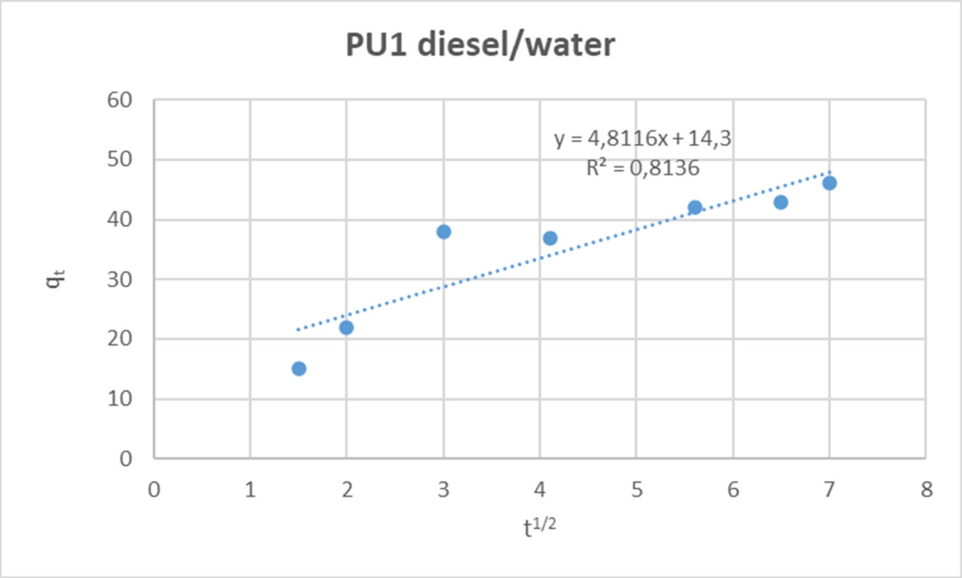

Figure S7. Intra-particle diffusion plot for PU1 in diesel/water.

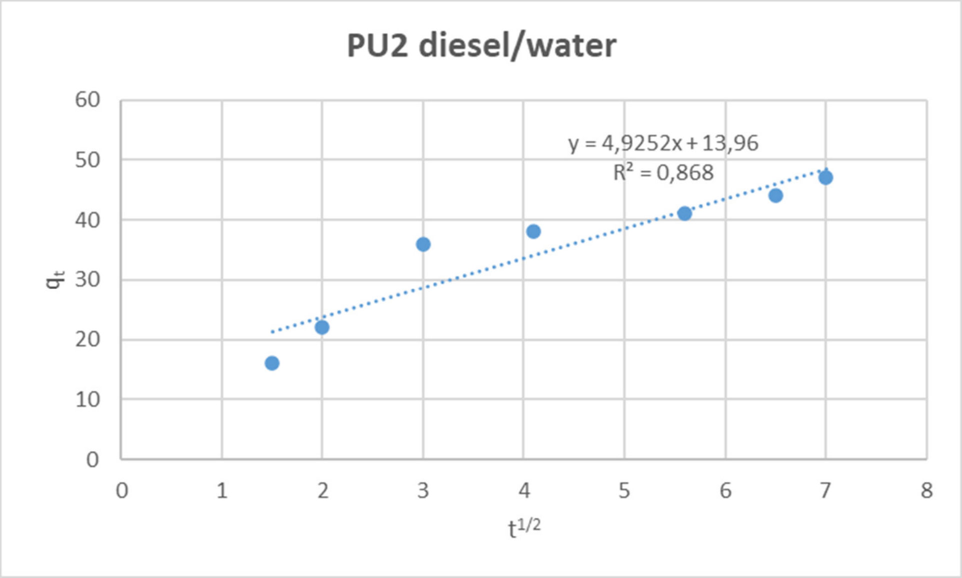

Figure S8. Intra-particle diffusion plot for PU2 in diesel/water.
